# Factors associated with postoperative physiological and psychological changes and rehabilitation exercise adherence in pediatric kidney transplant recipients

**DOI:** 10.3389/fpsyg.2026.1867348

**Published:** 2026-08-07

**Authors:** Zhaoguang Shang, Zhou Li

**Affiliations:** 1 Department of Physical Education, Zhongyuan University of Technology, Zhengzhou, China; 2 Department of Kidney Transplantation, The First Affiliated Hospital of Zhengzhou University, Zhengzhou, China

**Keywords:** rehabilitation exercise adherence, pediatric kidney transplantation, psychological status, quality of life, influencing factors

## Abstract

**Introduction:**

Postoperative rehabilitation exercise adherence is critical for recovery after pediatric kidney transplantation, yet its associated psychosocial factors remain insufficiently characterized. This study aimed to examine postoperative psychological status and rehabilitation exercise adherence in pediatric kidney transplant recipients, identify distinct adherence patterns, and determine associated influencing factors to inform targeted intervention strategies.

**Methods:**

A longitudinal study was conducted among 158 pediatric kidney transplant recipients (101 boys and 57 girls; mean age, 11.91 years; SD, 3.70) who underwent kidney transplantation between May 2008 and May 2024. Data were collected at 1, 2, 4, and 6 months postoperatively using the Rehabilitation Training Adherence Scale, the Pediatric Quality of Life Inventory 4.0 Transplant Module, and the Strengths and Difficulties Questionnaire. Latent class growth modeling and growth mixture modeling were used to identify adherence patterns. Group differences were examined using χ² tests and analyses of variance, and associated predictors were identified using multivariate logistic regression analyses.

**Results:**

Three distinct rehabilitation exercise adherence patterns were identified: a high adherence improvement–maintenance pattern (41.8%), a Moderate adherence with early improvement and slight decline pattern (29.7%), and a low adherence–stable pattern (28.5%). These patterns demonstrated substantial heterogeneity in postoperative rehabilitation exercise adherence among pediatric kidney transplant recipients.

**Discussion:**

Rehabilitation exercise adherence after pediatric kidney transplantation follows distinct longitudinal patterns and is influenced by clinical, demographic, and psychosocial factors. Early identification of recipients at risk of persistently poor adherence may facilitate targeted interventions to improve rehabilitation participation, quality of life, and psychological well-being, thereby promoting better postoperative recovery in this vulnerable population.

## 1 Introduction

Pediatric kidney transplantation is an effective treatment for children with uremia. It significantly prolongs their lives, supports their growth and development, and improves their quality of life, bringing it closer to that of healthy children. In recent years, the field of pediatric kidney transplantation in China has advanced rapidly, driven by national economic growth, improved medical conditions, and increased awareness, particularly through post-mortem organ donation programs. According to data from the National Kidney Disease Quality Control Center in 2025, the total number of dialysis patients in China reached 1.183 million. Although children comprise less than 1% of these patients, their number ranks second globally. After kidney transplantation, recipients experience significantly prolonged survival, with marked improvements in growth, development, and cognitive abilities. However, lifelong medication, sudden complications, and physical and mental discomforts, such as fatigue, headaches, and anxiety, reduce these children’s quality of life. Furthermore, the prolonged illness and the inability to engage in normal social activities severely affect their psychological wellbeing (Uçgun and Akgün Çıtak, 2024). Although numerous studies have been conducted on the postoperative survival rates and complications of pediatric kidney transplant recipients, both domestically and internationally, research on psychological state and adherence to rehabilitation exercises remains limited. This study aims to assess the psychological state and adherence to rehabilitation exercises in pediatric kidney transplant recipients, analyze their influencing factors, and provide a basis for developing intervention strategies.

## 2 Methods

### 2.1 Participants

This questionnaire-based longitudinal study was conducted among pediatric kidney transplant recipients treated at the Department of Kidney Transplantation, the First Affiliated Hospital of Zhengzhou University, between May 2008 and May 2024. The study aimed to examine postoperative physiological and psychological changes and rehabilitation exercise adherence in this population. Participants were eligible for inclusion if they were aged 3–18 years at the time of kidney transplantation and were able to complete postoperative follow-up assessments. The exclusion criteria were as follows: the presence of major diseases involving other vital organs, such as the heart or lungs; a documented mental illness that could interfere with questionnaire completion; a history of second kidney transplantation; major post-transplant organ disease; or refusal to participate in follow-up. Written informed consent was obtained from the participants’ parents or legal guardians, and assent was obtained from children when appropriate. The study was conducted in accordance with relevant medical ethics regulations.

### 2.2 Instruments

#### 2.2.1 Demographic and clinical variables

Demographic and clinical information was obtained from medical records and structured follow-up records. The variables included age, sex, only-child status, family structure, family income, postoperative body mass index, pre-transplant dialysis duration, dialysis modality, time from primary disease diagnosis to transplantation, post-transplant follow-up duration, and post-transplant complications.

#### 2.2.2 Rehabilitation training adherence scale

Rehabilitation exercise adherence was assessed using a validated Rehabilitation Training Adherence Scale (Frost et al., 2017). The scale consists of 20 items across four domains: physical exercise adherence, adherence to exercise-effect monitoring, proactive advice-seeking adherence, and adherence to exercise precautions. Responses were recorded on a 5-point Likert scale, and total scores were calculated according to the standard scoring procedure. Higher scores indicated greater adherence to rehabilitation exercise recommendations. In the present study, the scale demonstrated good internal consistency, with a Cronbach’s *α* coefficient of 0.865.

#### 2.2.3 Pediatric quality of life scale

Quality of life was assessed using the Pediatric Quality of Life Inventory 4.0 (Varni and Uzark, 2024). The instrument consists of four domains and 21 items assessing medication use, Physiological function, emotional function, social function, school performance. Responses were rated on a 5-point Likert scale and then reverse-scored and linearly transformed to a 0–100 scale, with scores of 0, 1, 2, 3, and 4 corresponding to 100, 75, 50, 25, and 0, respectively. Higher scores indicated better quality of life. Domain scores were calculated by summing the item scores within each domain and dividing the sum by the number of completed items. The total score was calculated as the mean of all completed item scores. The Chinese version of the instrument has demonstrated excellent internal consistency in previous validation studies, with a Cronbach’s *α* coefficient of 0.91.

#### 2.2.4 Strengths and difficulties questionnaire

Psychological status was assessed using the parent-report version of the Strengths and Difficulties Questionnaire, originally developed by Goodman in 1997 (Peng et al., 2026). The questionnaire consists of 25 items grouped into five subscales: emotional symptoms, conduct problems, hyperactivity/inattention, peer relationship problems, and prosocial behavior. The total difficulties score was calculated as the sum of the scores from the emotional symptoms, conduct problems, hyperactivity/inattention, and peer relationship problems subscales. Higher total difficulties scores indicated greater psychological difficulties, whereas higher prosocial behavior scores indicated stronger social functioning and prosocial skills. The Chinese version of the instrument has demonstrated good internal consistency, with a Cronbach’s *α* coefficient of 0.85.

### 2.3 Procedure

Clinical and demographic data were collected within 24 h of admission. After postoperative medical stability was confirmed by the transplant team, participants began a standardized, age-adapted rehabilitation exercise programme, generally starting 24–48 h after kidney transplantation and continuing for 6 months. Exercise prescriptions were individualized according to age, developmental stage, preoperative physical condition, graft function, wound healing, postoperative complications and exercise tolerance.

During hospitalization, rehabilitation focused on progressive low-intensity mobilization, including breathing exercises, ankle-pump and range-of-motion exercises, bed mobility, sitting, standing and walking, under the supervision of trained nurses or rehabilitation staff. After discharge, participants followed a progressive home-based programme comprising aerobic activity, flexibility exercises and, after adequate wound healing, light resistance exercises. Exercise type, duration and frequency were adapted for children aged 3–6, 7–12 and 13–18 years, and were progressed according to clinical stability and individual tolerance. Heavy lifting, substantial abdominal strain, contact sports and activities associated with graft-site trauma were avoided unless specifically approved by the transplant team. The complete age-specific exercise protocol, progression criteria, safety precautions and recommended activities are provided in Supplementary materials.

Before discharge, participants and their guardians received standardized verbal instructions and an illustrated exercise guide. Guardians supervised younger children, whereas older children and adolescents were encouraged to participate in exercise planning and self-monitoring. Participants recorded exercise type, frequency, approximate duration, missed sessions and exercise-related symptoms. Exercise was suspended or modified in the presence of acute infection, suspected rejection, unstable graft function, uncontrolled hypertension, postoperative complications or warning symptoms, including fever, chest pain, marked dyspnoea, unusual fatigue, gross haematuria or graft-site pain.

Follow-up assessments were conducted at 1, 2, 4 and 6 months after transplantation. At each assessment, researchers reviewed exercise completion, adherence to the prescribed frequency and duration, self-monitoring records, exercise-related barriers and help-seeking behaviours, consistent with the behavioural domains of the Rehabilitation Training Adherence Scale. Rehabilitation exercise adherence, health-related quality of life and psychological status were assessed using the Rehabilitation Training Adherence Scale, the Pediatric Quality of Life Inventory 4.0 and the Strengths and Difficulties Questionnaire, respectively. Exercise prescriptions were adjusted when poor tolerance, postoperative complications or difficulty completing the programme was reported.

Questionnaires were completed during outpatient visits or through standardized remote interviews conducted by trained researchers. Standardized instructions were provided before each assessment, and data confidentiality was emphasized. Each assessment required approximately 15–20 min. Completed questionnaires were checked for missing or internally inconsistent responses, and questionnaires with substantial missing data or uninterpretable responses were excluded from the analysis.

### 2.4 Data analysis

Total scores and domain scores were calculated for the Rehabilitation Training Adherence Scale, the Pediatric Quality of Life Inventory 4.0, and the Strengths and Difficulties Questionnaire to evaluate rehabilitation exercise adherence, quality of life, and psychological status among pediatric kidney transplant recipients. Statistical analyses were performed using SPSS version 24.0. Continuous variables were compared using analysis of variance, and categorical variables were compared using the *χ*^2^ test, as appropriate. Univariate and multivariable analyses were performed to identify factors associated with postoperative quality of life and psychological status. The candidate variables included sex, age at transplantation, postoperative body mass index, primary disease, pre-transplant dialysis duration, pre-transplant dialysis modality, follow-up duration, and post-transplant complications. Logistic regression models were used to examine factors associated with postoperative quality of life and psychological status. To identify longitudinal trajectories of rehabilitation exercise adherence, unconditional latent class growth models were constructed. The number of trajectory classes was increased sequentially, and model fit was compared across candidate models. Model selection was based on the Akaike information criterion, Bayesian information criterion, sample-size-adjusted Bayesian information criterion, entropy, and the bootstrap likelihood ratio test. Lower values of the information criteria indicated better model fit, whereas higher entropy indicated better classification accuracy. Participants were assigned to the trajectory class with the highest posterior probability.

## 3 Results

### 3.1 Demographic and clinical characteristics

Among 158 pediatric kidney transplant recipients, the median age was 13 years (IQR, 9–15), and 102 participants were male (64.6%). The mean BMI-for-age z-score (BAZ) was −0.94 (SD, 1.41). Among participants with available dialysis-duration data, 104 patients (68.4%) had undergone dialysis for ≤18 months and 48 (31.6%) for >18 months. Peritoneal dialysis was the most common dialysis modality (81/158, 51.3%), followed by hemodialysis (54/158, 34.2%). Postoperative complications were recorded in 32 patients (20.3%). Most participants were only children (92/158, 58.2%) and lived in nuclear families (104/158, 65.8%).

### 3.2 Postoperative rehabilitation training adherence, quality of life, and psychological status in pediatric kidney transplant patients at different time points

As shown in Table 1, rehabilitation exercise adherence scores increased from 73.34 ± 11.82 at 1 month post-transplantation to 83.08 ± 15.34 at 2 months, followed by a slight decrease at 4 months 82.26 ± 15.75 and 6 months 81.87 ± 15.76. Quality of life scores showed a continuous upward trend over time, increasing from 37.09 ± 11.90 at 1 month to 80.25 ± 17.95 at 6 months post-transplantation. Psychological status scores decreased from 29.06 ± 4.68 at 1 month to 26.83 ± 4.79 at 4 months, with a slight increase to 27.37 ± 4.35 at 6 months. Overall, pediatric kidney transplant recipients showed improved rehabilitation exercise adherence and quality of life during the postoperative follow-up period, while psychological difficulties tended to decrease over time.

**Table 1:** Scores of rehabilitation exercise adherence, quality of life, and psychological status at different postoperative time points in pediatric kidney transplant patients score, **x̄** ± s.

time	Rehabilitation exercise adherence	Quality of life	Psychological condition
One month post-surgery	73.34 ± 11.82	37.09 ± 11.90	29.06 ± 4.68
Two month post-surgery	83.08 ± 15.34	42.35 ± 10.25	28.37 ± 4.37
Four month post-surgery	82.26 ± 15.75	66.68 ± 19.72	26.83 ± 4.79
Six month post-surgery	81.87 ± 15.76	80.25 ± 17.95	27.37 ± 4.35

### 3.3 Trajectory of changes in postoperative functional training adherence in pediatric kidney transplant patients

As shown in Table 2, among 158 pediatric kidney transplant recipients with complete rehabilitation adherence data at 1, 2, 4 and 6 months after transplantation, latent class growth modeling identified three clinically interpretable adherence trajectories. The three-class model showed good classification quality, with an entropy of 0.924 and high average posterior probabilities across classes. The largest subgroup was characterized by consistently high adherence with further improvement after transplantation and subsequent maintenance of high scores, comprising 66 patients (41.8%). Their adherence scores increased from 83.64 ± 3.61 at 1 month to 96.12 ± 7.58 at 2 months, and remained high at 4 months and 6 months. The second subgroup included 47 patients (29.7%) and showed moderate adherence at 1 month, followed by improvement at 2 months and a mild decline thereafter. Their scores were 72.23 ± 6.44, 83.11 ± 9.91, 79.15 ± 7.09 and 78.57 ± 7.47 at 1, 2, 4 and 6 months, respectively. The third subgroup comprised 45 patients (28.5%) and was characterized by persistently lower adherence, with scores of 58.80 ± 7.53 at 1 month, 64.93 ± 6.85 at 2 months, 62.42 ± 7.39 at 4 months and 64.04 ± 11.59 at 6 months.

**Table 2:** Trajectory of changes in postoperative functional exercise adherence in pediatric kidney transplant recipients.

Project	Category	AIC	BIC	Entropy	Likelihood ratio test (*p*-value)		Category probability (%)
					LRT	BLRT	
Latent class growth model	1	5,210.513	5,222.764		100		
	2	4,797.179	4,821.68	0.912	<0.001	0.010	44.3
	3	4,672.4	4,709.151	0.924	<0.001	0.010	28.5
	4	4,600.529	4,649.531	0.966	<0.001	0.010	3.8
	5	4,567.079	4,628.331	0.974	<0.001	0.168	0.6

Overall, the three-class solution provided the most balanced and clinically interpretable classification of postoperative rehabilitation exercise adherence trajectories. These trajectories may reflect distinct patterns of postoperative behavioural adaptation and could help identify children requiring intensified rehabilitation guidance and follow-up support.

As shown in Table 3, rehabilitation exercise adherence scores differed significantly among the three trajectory groups across postoperative follow-up. The high adherence improvement–maintenance trajectory group had the highest adherence scores at all-time points, increasing from 83.64 ± 3.61 at 1 month post-transplantation to 96.12 ± 7.58 at 2 months, and remaining at a high level at 4 months (97.52 ± 3.27) and 6 months (96.44 ± 4.77). The moderate adherence with early improvement and slight decline group showed an increase from 72.23 ± 6.44 at 1 month to 83.11 ± 9.91 at 2 months, followed by a slight decline at 4 months (79.15 ± 7.09) and 6 months (78.57 ± 7.47). The low adherence–stable trajectory group consistently showed the lowest adherence scores, with only modest fluctuations over time. Repeated-measures analysis showed significant effects of group, time, and group-by-time interaction on rehabilitation exercise adherence scores (*F*_group_ = 886.78, *p* < 0.001; *F*_time_ = 73.45, *p* < 0.001; *F*_interaction_ = 6.93, *p* < 0.001), indicating that adherence levels varied across groups and changed differently over time.

**Table 3:** Comparison of rehabilitation exercise adherence scores at different time points after surgery among three groups of pediatric kidney transplant patients.

Group	Number	One month post-surgery	Two month post-surgery	Four month post-surgery	Six month post-surgery
High adherence improvement-maintenance trajectory	66 (41.8%)	83.64 ± 3.61	96.12 ± 7.58	97.52 ± 3.27	96.44 ± 4.77
Moderate adherence with early improvement and slight decline	47 (29.7%)	72.23 ± 6.44	83.11 ± 9.91	79.15 ± 7.09	78.57 ± 7.47
Low adherence-stable trajectory	45 (28.5%)	58.80 ± 7.53	64.93 ± 6.85	62.42 ± 7.39	64.04 ± 11.59

*F*_groups_ = 886.78, *p* < 0.001, *F*_Time_ = 73.45, *p* < 0.001, *F*_Interaction_ = 6.93, *p* < 0.001.

### 3.4 Univariate analysis of factors influencing changes in postoperative rehabilitation training adherence trajectories in pediatric kidney transplant patients

The high adherence improvement–maintenance group included 66 children, representing 41.8% of the study population. The mean age at kidney transplantation was 11.7 ± 3.7 years; 59.1% of participants were male, and 71.2% lived in nuclear families. The mean pre-transplant dialysis duration was 15.2 ± 13.3 months, and peritoneal dialysis was the most common dialysis modality (34/66, 51%). Postoperative complications occurred in 12 children (18.2%). The mean BMI-for-age z-score (BAZ) at transplantation was −0.86 ± 1.35. Rehabilitation exercise adherence increased markedly from 83.64 at 1 month to 96.12 at 2 months after transplantation and remained consistently high at 4 and 6 months, with mean scores of 97.52 and 96.44, respectively. This trajectory was characterized by early improvement followed by sustained high adherence, representing the most favourable rehabilitation exercise adherence pattern identified in this cohort.

The moderate adherence with early improvement and slight decline group included 47 children, representing 29.7% of the study population. The mean age at kidney transplantation was 11.9 ± 3.7 years; 70.2% of participants were male, and 63.8% lived in nuclear families. The mean pre-transplant dialysis duration was 17.1 ± 12.1 months, and peritoneal dialysis was the most common dialysis modality (27/47, 57.4%). Postoperative complications occurred in six children (12.8%). The mean BMI-for-age z-score (BAZ) at transplantation was −0.76 ± 1.52. Rehabilitation exercise adherence increased from 72.23 at 1 month to 83.11 at 2 months after transplantation, but then declined slightly to 79.15 at 4 months and 78.57 at 6 months. This trajectory was characterized by early improvement that was not fully sustained during follow-up, suggesting the need for continued monitoring and reinforcement of rehabilitation exercise behaviours in this subgroup.

The low adherence–stable group included 45 children, representing 28.5% of the study population. The mean age at kidney transplantation was 12.1 ± 3.8 years; 66.7% of participants were male, and 60.0% lived in nuclear families. The mean pre-transplant dialysis duration was 14.7 ± 12.6 months, and peritoneal dialysis was the most common dialysis modality (20/45, 44.4%). Postoperative complications occurred in nine children (20.0%). The mean BMI-for-age z-score (BAZ) at transplantation was −1.24 ± 1.35 among participants with valid BAZ data. Rehabilitation exercise adherence was low at 1 month after transplantation, with a mean score of 58.80, and increased only modestly to 64.93 at 2 months. Mean adherence scores remained low at 4 and 6 months, at 62.42 and 64.04, respectively. This trajectory was characterized by persistently low rehabilitation exercise adherence throughout follow-up, indicating sustained difficulties in establishing and maintaining regular rehabilitation exercise behaviours. Children in this subgroup may require early identification, individualized education, closer follow-up and continued family-supported interventions (Table 4).

**Table 4:** Univariate analysis of the trajectories influencing postoperative rehabilitation exercise adherence in pediatric kidney transplant patients.

Group	No.	The low adherence–stable group (*n* = 45)	Moderate adherence with early improvement and slight decline group (*n* = 47)	High adherence improvement–maintenance group (*n* = 66)	*χ* ^2^	*p*
Quality of life		82.07 ± 12.79	83.55 ± 11.06	93.41 ± 8.88	30.46	<0.001
Psychological condition		28.36 ± 3.60	28.70 ± 3.64	28.04 ± 3.12	22.1	<0.001
Gender					1.61	0.448
Male	30 (66.7)	33 (70.2)	39 (59.1)	30 (66.7)		
Female	15 (33.3)	14 (29.8)	27 (40.9)	15 (33.3)		
Transplantation age					64.5	<0.001
≤10	16 (35.6)	16 (34.0)	26 (39.4)	16 (35.6)		
>10	29 (64.4)	31 (66.0)	40 (60.6)	29 (64.4)		
BAZ					14.8	<0.01
<−2	34 (21.5)	12(25.0)	11 (23.4)	11 (16.7)		
≥ − 2	124 (79.5)	33 (75.0)	36 (76.6)	55 (83.3)		
Dialysis duration					39.8	<0.001
≤18月	29 (67.4)	29 (63.0)	46 (73.0)	29 (67.4)		
>18月	14 (32.6)	17 (37.0)	17 (27.0)	14 (32.6)		
Types of dialysis					28.1	<0.001
Hemodialysis	17 (37.8)	16 (34.0)	21 (31.8)	17 (37.8)		
Peritoneal dialysis	20 (44.4)	27 (57.4)	34 (51.5)	20 (44.4)		
Hemodialysis and peritoneal dialysis	1 (2.2)	3 (6.4)	3 (4.5)	1 (2.2)		
Other	7 (15.6)	1 (2.1)	8 (12.1)	7 (15.6)		
Postoperative complications					37.5	<0.001
No	36 (80.0)	41 (87.2)	49 (74.2)	36 (80.0)		
Yes	9 (20.0)	6 (12.8)	17 (25.8)	9 (20.0)		
Only child					13.1	0.05
Yes	26 (57.8)	31 (66.0)	35 (53.0)	26 (57.8)		
No	19 (42.2)	16 (34.0)	31 (47.0)	19 (42.2)		
Family structure					38.6	<0.001
Nuclear family	27 (60.0)	30 (63.8)	47 (71.2)	27 (60.0)		
Extended family	15 (33.3)	13 (27.7)	17 (25.8)	15 (33.3)		
Single-parent family	3 (6.7)	4 (8.5)	2 (3.0)	3 (6.7)		
Monthly income					20.7	<0.001
6,000	9 (20.0)	19 (40.4)	22 (33.3)	9 (20.0)		
6,000–12,000	21 (46.7)	20 (42.6)	31 (47.0)	21 (46.7)		
>12,000	15 (33.3)	8 (17.0)	13 (19.7)	15 (33.3)		

In the “Other” category of dialysis types, the expected frequency was less than 5; therefore, Fisher’s exact test was used, and the corresponding approximate *χ*^2^ value is reported.

### 3.5 Multifactorial analysis of factors influencing postoperative psychological status and rehabilitation training adherence in pediatric kidney transplant recipients

Logistic regression analysis was conducted with children’s quality of life, psychological state, age, postoperative BAZ, dialysis duration, type of dialysis, postoperative complications, only-child status, family composition, and family monthly income as independent variables, using the categorical analysis of rehabilitation exercise adherence changes as the dependent variable, with the consistently high adherence group as the control. The results showed that the core risk factors for the moderately declining adherence group were: long dialysis duration, hemodialysis, postoperative complications, single-parent family, and low income, while the protective factors included: older age and good baseline quality of life. Children in this group experienced a significant decline in adherence over time, due to a heavy clinical burden and insufficient socioeconomic support. The core risk factors for the low-adherence-improving group were: younger age, single-parent family, and low income, while preoperative clinical factors (dialysis duration, complications) had no significant effect. This suggests that adherence in this group was mainly influenced by family socioeconomic status and age, with poor early adherence but improvement over time (Table 5).

**Table 5:** Multivariate analysis of factors affecting trajectory categories of postoperative rehabilitation exercise adherence in pediatric kidney transplant recipients (*n* = 158).

Dependent variable	Independent variable	*B*	SE	Wald *χ*^2^	OR (95% CI)	*p*
Moderate adherence with early improvement and slight vs High adherence improvement-maintenance trajectory	Quality of life	−0.12	0.03	16	0.89 (0.84–0.94)	<0.001
	Age (Y)	−0.28	0.09	9.7	0.76 (0.64–0.90)	0.002
	BAZ	−0.45	0.26	2.82	0.63 (0.37–1.07)	0.093
	Dialysis duration (M)	0.21	0.05	17.6	1.23 (1.12–1.36)	<0.001
	Types of dialysis (control = peritoneal dialysis)					
	Hemodialysis	0.96	0.38	6.4	2.61 (1.24–5.50)	0.011
	Hemodialysis and peritoneal dialysis	0.52	0.62	0.7	1.68 (0.50–5.66)	0.401
	Other	0.33	0.81	0.2	1.39 (0.28–6.85)	0.684
	Postoperative complications (yes vs. no)	1.83	0.46	15.8	6.23 (2.53–15.3)	<0.001
	Only child (yes vs. no)	−0.58	0.41	2	0.56 (0.25–1.25)	0.157
	Family structure(control = nuclear family)					
	Extended family	0.62	0.44	2	1.86 (0.79–4.38)	0.158
	Single-parent family	1.28	0.51	6.3	3.60 (1.32–9.81)	0.012
	Monthly income (control = >12,001)					
	<6,000元	1.16	0.52	5	3.19 (1.15–8.84)	0.026
	6,001–12,000元	0.53	0.48	1.2	1.70 (0.66–4.37)	0.271
Low adherence-stable trajectory vs High adherence improvement-maintenance trajectory	Quality of life	−0.18	0.04	20.3	0.84 (0.78–0.90)	<0.001
	Age (Y)	−0.65	0.12	29.4	0.52 (0.41–0.66)	<0.001
	BAZ	0.106	0.16	9.3	1.11 (0.64–0.91)	<0.001
	Dialysis duration (M)	0.09	0.06	2.3	1.09 (0.97–1.23)	0.13
	Types of dialysis (control = peritoneal dialysis)					
	Hemodialysis	−0.45	0.51	0.8	0.64 (0.24–1.73)	0.378
	Hemodialysis and peritoneal dialysis	−0.21	0.76	0.1	0.81 (0.18–3.60)	0.782
	Other	0.38	0.89	0.2	1.46 (0.25–8.42)	0.669
	Postoperative complications (yes vs. no)	0.42	0.59	0.5	1.52 (0.48–4.81)	0.477
	Only child (yes vs. no)	−0.91	0.48	3.6	0.40 (0.16–1.03)	0.058
	Family structure (control = nuclear family)					
	Extended family	0.44	0.58	0.6	1.55 (0.50–4.84)	0.448
	Single-parent family	2.21	0.55	16.1	9.12 (3.10–26.8)	<0.001
	Monthly income (control = >12,001)					
	<6,000元	1.62	0.61	7.1	5.05 (1.53–16.7)	0.008
	6,001–12,000元	0.78	0.58	1.8	2.18 (0.70–6.79)	0.18

## 4 Discussion

### 4.1 Postoperative rehabilitation training adherence in pediatric kidney transplant patients is at a medium-to-high level

After kidney transplant surgery, appropriate and progressive functional training can enhance muscle endurance and promote cardiopulmonary function recovery in pediatric patients. Studies have shown a positive correlation between adherence to postoperative functional training and renal function recovery. As adherence increases, the risk of postoperative complications decreases, and renal function gradually improves. Adherence directly influences the effectiveness of postoperative functional training. Moreover, postoperative rehabilitation exercises for kidney transplant patients require long-term commitment. During hospitalization, in-hospital rehabilitation therapists provide guidance, and with the attending physician’s approval, patients are usually required to continue functional training for at least 6–12 months post-surgery. Therefore, patients’ adherence to medical advice and active participation in the postoperative rehabilitation plan are crucial for reducing complication rates, promoting renal recovery, and improving postoperative quality of life. In this study, adherence to postoperative rehabilitation exercises among kidney transplant patients was at a moderate level, similar to the adherence score of (70.07 ± 6.32) reported by Du Huili. This may be because the study included patients with the same type of disease. Although there were concerns about the need for long-term functional exercises after surgery, overall adherence remained at a moderate level, considering the recovery of kidney function. However, the adherence to postoperative rehabilitation exercises in these patients still needs improvement, indicating that the issue of rehabilitation exercise adherence in kidney transplant patients requires continued attention from medical staff.

### 4.2 Trajectory of changes in postoperative rehabilitation training adherence in pediatric kidney transplant patients

This study identified three trajectories of postoperative rehabilitation exercise adherence in pediatric kidney transplant patients: a consistently high adherence group, a moderately declining adherence group, and a low adherence increasing group. This confirms significant differences in rehabilitation exercise adherence among pediatric kidney transplant patients at various postoperative time points. Among these three trajectories, children in the consistently increasing adherence group showed relatively good rehabilitation exercise adherence at 1 month postoperatively, with a gradual upward trend over 6 months. This indicates that these patients have good postoperative rehabilitation exercise adherence and are able to gradually adapt to and cooperate with postoperative functional training, thereby improving their adherence over time. It is recommended that healthcare professionals conduct accurate postoperative assessments and implement timely, appropriate interventions to promote improved rehabilitation exercise adherence and facilitate early recovery.

The number of patients with declining adherence was relatively small. Most pediatric patients maintained moderate adherence to rehabilitation exercises within 6 months post-surgery, with a gradual increase or decrease at different time points. This suggests that although postoperative functional training adherence is a long-term and challenging process to maintain, most kidney transplant patients can still sustain a certain level of exercise adherence. It is recommended that healthcare professionals encourage these patients and help them maintain and improve their adherence levels.

This study also found that children in the low adherence improvement group had moderate adherence to rehabilitation exercises 1 month after surgery, with overall adherence initially low but gradually increasing over time. The low adherence in these patients may result from failure to master correct exercise methods before and after starting the rehabilitation programme, compounded by physiological factors such as discomfort and negative emotions during exercise, leading to relatively low adherence to physical function training. This suggests that these patients are significantly affected by factors related to rehabilitation treatment. Medical staff need to strengthen guidance on rehabilitation exercises, promptly identify patients with declining adherence, help them quickly master correct exercise methods, and alleviate negative emotions to improve adherence.

### 4.3 Factors influencing postoperative rehabilitation training adherence in pediatric kidney transplant patients

#### 4.3.1 Age and BMI-for-age z-score (BAZ)

Studies have shown that older children, especially those in the high-adherence group with an average age of 14.2 years, better understand the benefits of exercise, are more self-disciplined, and require less parental supervision. As a result, they are more likely to consistently maintain their exercise routines. Younger children, particularly those in the low-adherence-to-compensation group with an average age of only 8.5 years, are more likely to face difficulties with monotonous rehabilitation exercises and struggle to remember complex movements. Their initial low adherence is not due to a lack of desire to improve, but rather because they “cannot do” or “cannot remember.” (Elshafey et al., 2022) Consistent with international research findings, this study also found that BMI-for-age z-score (BAZ) affects the quality of life in pediatric kidney transplant recipients. Before kidney transplantation, children with uremia generally have poor nutritional status. Postoperatively, with improvements in the internal environment and the recovery of various systems, children can achieve a normal body mass index, thereby improving their quality of life through a well-planned diet (Beladi Mousavi et al., 2020).

#### 4.3.2 Dialysis time and complications

The longer the dialysis duration and the more postoperative complications, the more difficult it is for children to maintain their exercise routines. This “giving up” does not typically happen immediately but occurs gradually over time (Karaterzi et al., 2025). Children with shorter dialysis durations (≤18 months) generally have better physical health, along with the energy and stamina to complete their daily exercise tasks. As a result, they are more likely to be part of the “highly compliant group.” On the other hand, children with longer dialysis durations (>18 months) experience significant physical strain due to prolonged dialysis, and many suffer from anemia, malnutrition, and muscle atrophy (Kogon and Harshman, 2019). Long-term dialysis also habituates children to the “patient” role, making it difficult to transition from a relatively sedentary lifestyle to an “active exercise mode” in the short term.

Postoperative complications, including rejection, lung infections, and hypertension, significantly affect rehabilitation outcomes. Children without complications tend to have a smooth postoperative recovery, with minimal discomfort. Consequently, they have the motivation and energy to exercise, which makes it easier for them to maintain long-term adherence. In contrast, complications can cause children to feel weak and fatigued. Treatment for rejection often requires higher medication dosages, and the side effects of these medications can exacerbate fatigue (Prudhomme et al., 2023).

Therefore, for children undergoing prolonged dialysis or experiencing postoperative complications, medical staff and parents must provide additional patience and support. These children cannot be expected to adapt as quickly as healthy children. The primary focus should be on improving nutrition to aid recovery, managing complications, and gradually encouraging exercise to prevent them from falling behind due to physical limitations.

#### 4.3.3 Family structure and household income

Children are more likely to adhere to exercise routines when they come from complete family structures and higher-income households. Conversely, children with less family support and greater financial hardship are more likely to fall behind. Family structures are categorized into three types: nuclear families (parents + child), extended families (grandparents + parents + child), and single-parent families (one parent raising the child alone). While children in extended families show slightly lower adherence compared to those in nuclear families, the difference is not statistically significant. This suggests that having “two or more adults in the family” acts as a protective factor. Children from single-parent families are 3.6 times more likely to fall into the “medium adherence decline group” (giving up midway) compared to children from nuclear families. Additionally, their risk of falling into the “low adherence rise group” (difficulty maintaining exercise from the start) is 9.12 times higher. This indicates that children in single-parent families find it more difficult to establish an exercise habit from the outset.

Higher-income families generally have more resources (Shingde et al., 2024). For instance, they can afford better nutritional supplements for their children, hire rehabilitation therapists, and parents have more time and energy (or can hire help to manage household tasks) to support their children. In contrast, lower-income families often face significant financial pressures due to high treatment costs. Parents, preoccupied with earning a living, may not fully recognize the long-term importance of rehabilitation exercises, resulting in insufficient adherence (Ramay et al., 2017). For these patients, medical staff can, within the scope of policy guidelines, select more appropriate treatment plans and, when necessary, seek assistance from charitable organizations to help alleviate their difficulties.

#### 4.3.4 Quality of life and psychological status score

The child’s early postoperative living conditions (quality of life) and psychological state (emotional behavior) serve as the “intrinsic motivation” that determines whether they can persist in exercise. The more comfortable and relaxed they are, the more proactive they will be in exercising. Conversely, the more physically uncomfortable and emotionally depressed they are, the more likely they are to give up on exercise (Marshall et al., 2024). Quality of life addresses the question of “can”—it determines the child’s physiological ability to exercise. The SDQ addresses the questions of “willingness” and “ability to persist” (Diseth et al., 2011), it determines the child’s psychological motivation and behavioural regulation ability to initiate and maintain exercise (Selewski et al., 2015).

In logistic regression, the odds ratios (OR) for both variables are between 0.8 and 0.9, meaning that with every unit of deterioration, the risk of the child falling into the “low adherence group” increases significantly. This suggests that adherence to rehabilitation exercises is not merely a medical issue, but also a socio-psychological issue that involves both the mind and body. If a child experiences physical discomfort after surgery (poor quality of life) and simultaneously faces high psychological stress and emotional instability (high SDQ score), they will almost inevitably fall into a low adherence situation. In contrast, only when a child is physically comfortable and mentally energetic can rehabilitation exercises truly become integrated into their life.

## 5 Limitations

Despite several strengths, this study has some limitations. First, the single-center design and limited sample size (*N* = 158) may restrict the generalizability of our findings to pediatric kidney transplant populations in other healthcare settings. Second, the follow-up period was limited to 6 months post-transplantation, preventing assessment of long-term adherence trajectories and their associations with later graft outcomes or psychosocial adjustment. Third, although validated instruments were used, all primary outcomes—including rehabilitation exercise adherence, quality of life, and psychological status—relied on self- or parent-reported measures, introducing potential informant and social desirability bias. Fourth, unmeasured confounding factors—such as pre-existing cognitive or developmental delays, parental mental health, health literacy, and patient–provider communication quality—may have influenced both adherence patterns and psychological outcomes but were not captured in this analysis. Fifth, the classification of adherence trajectories using latent class growth modeling, while methodologically robust, remains data-driven; replication in independent cohorts is needed to confirm the three-group solution. To address these limitations, future multi-center, long-term prospective studies employing objective adherence monitoring (e.g., wearable sensors or electronic logs) and comprehensive assessment of family and cognitive factors are warranted to further elucidate the dynamic interplay of clinical, psychological, and social determinants of rehabilitation adherence in this vulnerable population.

## 6 Conclusion

This longitudinal study identified three distinct trajectory categories of rehabilitation exercise adherence among pediatric kidney transplant recipients using a growth mixture model. The findings showed that children’s quality of life, psychological state, age, BMI-for-age z-score (BAZ), family structure, average monthly income per capita, dialysis duration, and postoperative complications were associated with different adherence trajectory categories.

The innovation of this study lies in its longitudinal assessment of dynamic changes in rehabilitation exercise adherence after pediatric kidney transplantation, rather than evaluating adherence at a single time point. By identifying heterogeneous adherence trajectories, this study provides a more detailed understanding of postoperative rehabilitation behavior in children with end-stage renal disease after kidney transplantation. These findings have important clinical implications. Healthcare professionals may use demographic and clinical characteristics, together with quality of life and psychological assessment results, to identify children at risk of poor rehabilitation exercise adherence. Early identification of low-adherence groups may help pediatric transplant teams provide more individualized follow-up, rehabilitation guidance, and psychological support.

Furthermore, intervention programs targeting modifiable factors such as quality of life and psychological state may help improve rehabilitation exercise adherence and promote early postoperative recovery. These results provide empirical evidence for rehabilitation psychologists and interdisciplinary pediatric transplant teams to develop individualized postoperative rehabilitation management strategies for this vulnerable population.

## Data Availability

The raw data supporting the conclusions of this article will be made available by the authors, without undue reservation.
