# Supplementary material for: Factors associated with postoperative physiological and psychological changes and rehabilitation exercise adherence in pediatric kidney transplant recipients
Source: Front Psychol. 2026 Aug 7;17:1867348. doi: 10.3389/fpsyg.2026.1867348 (PMC13494696; doi:10.3389/fpsyg.2026.1867348)
Supplement: Supplementary file 1 [file Supplementary_file_1.docx]

Children's Quality of Life Questionnaire (PedsQL™ 4.0 Parent-Agent Report Version)

Dear Participant:

Hello! We are researchers from Chengdu Medical College. To better provide targeted care and health guidance for pediatric kidney transplant patients, we are conducting a survey on "Analysis of Influencing Factors of Postoperative Physiological and Psychological Changes and Rehabilitation Exercise Compliance in Pediatric Kidney Transplant Patients." Completing this questionnaire will take approximately 15-20 minutes. There are no right or wrong answers; please answer truthfully based on your own situation. You may exit at any time if you feel uncomfortable during the survey. This questionnaire is anonymous, and we promise that the information you provide will be strictly confidential. The research results will only be used for scientific research. Thank you sincerely for your participation and support.

Instructions: Please evaluate your child based on their actual situation over the past month and mark the option that best matches your assessment with a "√".

|  |  | **Never** | **Almost**  **Never** | **Some-**  **times** | **Often** | **Almost**  **Always** |
| --- | --- | --- | --- | --- | --- | --- |
| 1 | Walking more than one block | □ | □ | □ | □ | □ |
| 2 | Running | □ | □ | □ | □ | □ |
| 3 | Participating in sports activity or exercise | □ | □ | □ | □ | □ |
| 4 | Lifting something heavy | □ | □ | □ | □ | □ |
| 5 | Taking a bath or shower by him or herself | □ | □ | □ | □ | □ |
| 6 | Doing chores around the house | □ | □ | □ | □ | □ |
| 7 | Having hurts or aches | □ | □ | □ | □ | □ |
| 8 | Low energy level | □ | □ | □ | □ | □ |
| 9 | Feeling afraid or scared | □ | □ | □ | □ | □ |
| 10 | Feeling sad or blue | □ | □ | □ | □ | □ |
| 11 | Feeling angry | □ | □ | □ | □ | □ |
| 12 | Worrying about what will happen to him or her | □ | □ | □ | □ | □ |
| 13 | Getting along with other children | □ | □ | □ | □ | □ |
| 14 | Other kids not wanting to be his or her friend | □ | □ | □ | □ | □ |
| 15 | Not able to do things that other children his or her age can do | □ | □ | □ | □ | □ |
| 16 | Keeping up when playing with other children | □ | □ | □ | □ | □ |
| 17 | Paying attention in class | □ | □ | □ | □ | □ |
| 18 | Forgetting things | □ | □ | □ | □ | □ |
| 19 | Keeping up with schoolwork | □ | □ | □ | □ | □ |
| 20 | Missing school because of not feeling well | □ | □ | □ | □ | □ |
| 21 | Missing school to go to the doctor or hospital | □ | □ | □ | □ | □ |
