# Supplementary material for: Factors associated with postoperative physiological and psychological changes and rehabilitation exercise adherence in pediatric kidney transplant recipients
Source: Front Psychol. 2026 Aug 7;17:1867348. doi: 10.3389/fpsyg.2026.1867348 (PMC13494696; doi:10.3389/fpsyg.2026.1867348)
Supplement: Supplementary file 2 [file Supplementary_file_2.docx]

# Rehabilitation Training Adherence Scale (RTAS)

## Participant Information Sheet

Dear Participant,

You are invited to participate in this survey assessing adherence to rehabilitation training. The purpose of this questionnaire is to understand how well individuals follow prescribed rehabilitation exercises and recommendations during the recovery process.

Your participation is entirely voluntary. All information collected will be used solely for scientific research purposes. The questionnaire is anonymous, and all responses will be kept strictly confidential. No personally identifiable information will be disclosed to any third party.

There are no right or wrong answers. Please respond honestly based on your actual experiences during your rehabilitation period.

Thank you for your valuable participation.

## Instructions

Please indicate how often each statement describes your actual behavior during your rehabilitation period.

### Response Options

| Score | Response |
| --- | --- |
| 1 | Never |
| 2 | Rarely |
| 3 | Sometimes |
| 4 | Often |
| 5 | Always |

# Section I. Rehabilitation Exercise Performance Adherence

| No. | Item | Never | Rarely | Sometimes | Often | Always |
| --- | --- | --- | --- | --- | --- | --- |
| 1 | I am able to understand and master the specific methods of rehabilitation training. | □ | □ | □ | □ | □ |
| 2 | I perform rehabilitation exercises every day according to healthcare professionals’ instructions. | □ | □ | □ | □ | □ |
| 3 | I am able to schedule a fixed time for rehabilitation exercises. | □ | □ | □ | □ | □ |
| 4 | I perform upper-limb resistance exercises every day. | □ | □ | □ | □ | □ |
| 5 | I perform lower-limb flexion and extension exercises every day. | □ | □ | □ | □ | □ |
| 6 | I perform deep-breathing exercises every day. | □ | □ | □ | □ | □ |
| 7 | I practice walking exercises every day. | □ | □ | □ | □ | □ |
| 8 | I strictly follow the prescribed number of repetitions in my rehabilitation program. | □ | □ | □ | □ | □ |
| 9 | I strictly follow the prescribed duration of rehabilitation exercises. | □ | □ | □ | □ | □ |
| 10 | I complete all exercises included in my rehabilitation program without omission. | □ | □ | □ | □ | □ |

# Section II. Self-Management and Monitoring Adherence

| No. | Item | Never | Rarely | Sometimes | Often | Always |
| --- | --- | --- | --- | --- | --- | --- |
| 11 | I continue rehabilitation exercises even when nobody reminds or supervises me. | □ | □ | □ | □ | □ |
| 12 | I actively monitor and evaluate whether my physical function is improving as expected. | □ | □ | □ | □ | □ |
| 13 | When family members or friends encourage me to exercise, I comply with their suggestions. | □ | □ | □ | □ | □ |
| 14 | I attend follow-up appointments as scheduled by my healthcare providers. | □ | □ | □ | □ | □ |

# Section III. Healthcare Communication Adherence

| No. | Item | Never | Rarely | Sometimes | Often | Always |
| --- | --- | --- | --- | --- | --- | --- |
| 15 | When I encounter problems that I or my family cannot solve during rehabilitation, I seek advice from healthcare professionals promptly. | □ | □ | □ | □ | □ |
| 16 | When my recovery does not meet the expected goals explained by healthcare professionals, I actively seek professional guidance. | □ | □ | □ | □ | □ |
| 17 | If I experience localized pain during rehabilitation exercises, I proactively consult healthcare professionals. | □ | □ | □ | □ | □ |
| 18 | If I experience swelling or other abnormal symptoms during rehabilitation exercises, I proactively consult healthcare professionals. | □ | □ | □ | □ | □ |

# Section IV. Safety Adherence

| No. | Item | Never | Rarely | Sometimes | Often | Always |
| --- | --- | --- | --- | --- | --- | --- |
| 19 | In daily life, I avoid exceeding the weight-bearing or exercise intensity limits prescribed by healthcare professionals. | □ | □ | □ | □ | □ |
| 20 | In daily life, I avoid potentially risky activities such as jumping, sudden turning, or abrupt stopping movements. | □ | □ | □ | □ | □ |

# Confidentiality Statement

All information provided in this questionnaire will be treated confidentially and used exclusively for research purposes. Individual responses will be anonymized and analyzed only in aggregate form. Participation is voluntary, and respondents may withdraw at any time without any consequences.
