# Supplementary material for: Factors associated with postoperative physiological and psychological changes and rehabilitation exercise adherence in pediatric kidney transplant recipients
Source: Front Psychol. 2026 Aug 7;17:1867348. doi: 10.3389/fpsyg.2026.1867348 (PMC13494696; doi:10.3389/fpsyg.2026.1867348)
Supplement: Supplementary file 3 [file Supplementary_file_3.docx]

# Strengths and Difficulties Questionnaire (SDQ)

## Participant Information Sheet

Dear Parent/Guardian,

You are invited to participate in this survey designed to assess children’s emotional and behavioral functioning. The purpose of this questionnaire is to better understand children’s psychological adjustment, social relationships, emotional well-being, and behavioral characteristics.

Your participation is entirely voluntary. All information collected will be used solely for scientific research purposes. The questionnaire is anonymous, and all responses will be kept strictly confidential. There are no right or wrong answers. Please answer each question based on your child’s behavior during the past six months.

Thank you for your participation and support.

## Instructions

Please read each statement carefully and indicate how well it describes your child during the past six months.

### Response Options

| Score | Response |
| --- | --- |
| 0 | Not True |
| 1 | Somewhat True |
| 2 | Certainly True |

# Strengths and Difficulties Questionnaire (SDQ)

| No. | Item | Not True | Somewhat True | Certainly True |
| --- | --- | --- | --- | --- |
| 1 | Considerate of other people’s feelings | □ | □ | □ |
| 2 | Restless, overactive, cannot stay still for long | □ | □ | □ |
| 3 | Often complains of headaches, stomachaches, or sickness | □ | □ | □ |
| 4 | Shares readily with other children (treats, toys, pencils, etc.) | □ | □ | □ |
| 5 | Often has temper tantrums or hot tempers | □ | □ | □ |
| 6 | Rather solitary, tends to play alone | □ | □ | □ |
| 7 | Generally obedient, usually does what adults request | □ | □ | □ |
| 8 | Has many worries, often seems worried | □ | □ | □ |
| 9 | Helpful if someone is hurt, upset, or feeling ill | □ | □ | □ |
| 10 | Constantly fidgeting or squirming | □ | □ | □ |
| 11 | Has at least one good friend | □ | □ | □ |
| 12 | Often fights with other children or bullies them | □ | □ | □ |
| 13 | Often unhappy, depressed, or tearful | □ | □ | □ |
| 14 | Generally liked by other children | □ | □ | □ |
| 15 | Easily distracted, concentration wanders | □ | □ | □ |
| 16 | Nervous or clingy in new situations, easily loses confidence | □ | □ | □ |
| 17 | Kind to younger children | □ | □ | □ |
| 18 | Often lies or cheats | □ | □ | □ |
| 19 | Picked on or bullied by other children | □ | □ | □ |
| 20 | Often volunteers to help others (parents, teachers, other children) | □ | □ | □ |
| 21 | Thinks things out before acting | □ | □ | □ |
| 22 | Steals from home, school, or elsewhere | □ | □ | □ |
| 23 | Gets along better with adults than with other children | □ | □ | □ |
| 24 | Has many fears, easily scared | □ | □ | □ |
| 25 | Sees tasks through to the end; has good attention span | □ | □ | □ |

# Confidentiality Statement

All information collected in this questionnaire will be treated as strictly confidential and used exclusively for research purposes. Individual responses will remain anonymous and will be analyzed only in aggregated form. Participation is voluntary, and participants may withdraw at any time without any negative consequences.

Thank you for your participation.
