# Supplementary material for: Factors associated with postoperative physiological and psychological changes and rehabilitation exercise adherence in pediatric kidney transplant recipients
Source: Front Psychol. 2026 Aug 7;17:1867348. doi: 10.3389/fpsyg.2026.1867348 (PMC13494696; doi:10.3389/fpsyg.2026.1867348)
Supplement: Supplementary file 4 [file Supplementary_file_4.docx]

**Supplementary Rehabilitation Exercise Protocol**

*Age-adapted postoperative rehabilitation for pediatric kidney transplant recipients*

# Supplementary Methods

# Age-adapted postoperative rehabilitation exercise protocol

## General principles

The rehabilitation programme was initiated after postoperative medical stability had been confirmed by the transplant team and continued for six months after kidney transplantation. The programme comprised four consecutive phases: the inpatient phase, the first 0–6 weeks after discharge, the transitional phase at 6–8 weeks and the maintenance phase from 8 weeks to 6 months.

Exercise prescriptions were individualized according to age, developmental stage, preoperative physical condition, graft function, wound healing, postoperative complications and exercise tolerance. Progression was determined by clinical stability and tolerance rather than postoperative time alone. Participants did not automatically progress to the next phase if wound healing was incomplete, graft function was unstable or exercise-related symptoms were present.

Before each exercise session, participants or their guardians checked for fever, symptoms of infection, dizziness, unusual fatigue, wound abnormalities, graft-site pain, gross haematuria and physician-directed activity restrictions. Each home-based session included a brief warm-up, the prescribed main activity and a cool-down period. Exercise type, frequency, approximate duration, missed sessions and exercise-related symptoms were documented in an exercise record.

Exercise intensity was primarily monitored using the talk test and a 0–10 rating of perceived exertion scale. Light-intensity activity corresponded approximately to a perceived exertion of 2–3 out of 10, whereas moderate-intensity activity corresponded to approximately 3–5 out of 10. During moderate-intensity activity, participants were expected to breathe more rapidly while remaining able to speak in complete sentences. For children aged 3–6 years who could not reliably report perceived exertion, intensity was assessed by the guardian on the basis of breathing pattern, facial expression, movement quality and ability to speak normally.

Participants were instructed to avoid breath-holding and the Valsalva manoeuvre during all activities. Heavy lifting, strenuous abdominal exercises, rough play, collision activities and direct pressure on the graft area were not included in the programme. Swimming was permitted only after complete wound healing and approval by the transplant team.

## Pre-exercise clinical assessment

Before commencing inpatient rehabilitation, the following conditions were confirmed by the transplant team:

1. Haemodynamic and respiratory stability.

2. Absence of active bleeding or acute surgical complications.

3. Adequate postoperative pain control.

4. Ability to follow simple exercise instructions, with guardian assistance when required.

5. No physician-directed requirement for strict bed rest.

6. No acute condition requiring suspension of physical activity.

Before progression to moderate-intensity aerobic activity or resistance exercise, participants were reassessed for wound healing, graft function, blood pressure control, infection, rejection, musculoskeletal limitations and exercise-related symptoms.

## Standard session structure

A typical exercise session comprised:

1. Pre-exercise screening: approximately 1–2 min.

2. Warm-up: 3–5 min of slow walking, gentle joint movement or age-appropriate movement games.

3. Main activity: according to postoperative phase and age group.

4. Cool-down: 3–5 min of gradually slower movement and gentle stretching.

5. Recording: documentation of activity type, duration, symptoms and reasons for any incomplete session.

Stretching was performed slowly and without bouncing. Each position was held for approximately 10–20 s according to age and tolerance. Movements causing abdominal pulling, wound discomfort or graft-site pressure were discontinued.

## Phase 1: inpatient rehabilitation

Inpatient rehabilitation generally commenced 24–48 h after transplantation once medical stability was confirmed. Initial sessions were conducted at the bedside under direct supervision.

The first level consisted of diaphragmatic breathing, ankle-pump exercises, active range-of-motion exercises, repositioning and assisted bed mobility. Participants performed approximately 5–10 diaphragmatic breaths, 10–20 ankle-pump repetitions and 5–10 repetitions of comfortable active movement for the major joints.

When these activities were tolerated, participants progressed to sitting at the edge of the bed, transferring to a chair, supported standing, repeated sit-to-stand movements and short-distance walking. Walking distance was increased from bedside activity to walking within the room and subsequently to corridor walking.

Activities were divided into short sessions to avoid excessive fatigue. Depending on clinical status, participants completed approximately two to four mobilization sessions per day. Younger children completed mobilization through play, reaching, standing games or walking with a guardian. Older children and adolescents were encouraged to participate actively in transfers and walking.

Progression during hospitalization required:

- Completion of the current activity without dizziness, marked dyspnoea, graft-site pain or wound discomfort.
- Stable clinical observations as judged by the clinical team.
- No loss of balance or deterioration in movement quality.
- Recovery to the pre-exercise condition shortly after the session.
- No worsening of symptoms later on the same day.

The participant returned to the previous tolerated level if symptoms developed or if postoperative status deteriorated.

## Phase 2: home-based rehabilitation during the first 0–6 weeks after discharge

The objectives during the first six weeks were to restore routine mobility, reduce prolonged sedentary behaviour and gradually improve walking tolerance while protecting the surgical wound and graft area.

Prescribed activities included:

- Slow or comfortable-paced walking.
- Gentle active range-of-motion exercises.
- Light flexibility exercises.
- Age-appropriate active play.
- Basic balance activities performed near stable support.
- Light daily activities that did not involve lifting, pushing, pulling or abdominal strain.

Exercise was initially accumulated in short bouts. Participants who could not complete the prescribed duration continuously divided the activity into two or three sessions during the day. Exercise duration was increased before exercise intensity.

During this phase, participants avoided running, jumping, vigorous cycling, abdominal strengthening, weighted resistance exercise, rough play and activities with a risk of falling or abdominal impact. Outdoor exercise was postponed during extreme weather, acute illness or conditions associated with dehydration risk.

Guardians directly supervised children aged 3–12 years. Adolescents could exercise independently in a safe environment after receiving instructions, but guardians reviewed exercise completion and symptoms. Exercise records were completed by guardians for younger children and jointly or independently by older children and adolescents.

## Phase 3: transitional rehabilitation at 6–8 weeks

At approximately 6 weeks, the exercise programme was reviewed. Progression was permitted only when the wound had healed adequately, graft function was clinically stable, blood pressure was controlled, no acute infection or suspected rejection was present and the participant had tolerated the preceding phase.

Aerobic exercise was gradually progressed from comfortable-paced walking to brisk walking, stationary cycling, low-impact movement games or other non-contact activities. Exercise remained predominantly light in intensity, with brief periods of moderate-intensity activity introduced according to tolerance.

Light muscle-strengthening exercises were introduced after medical clearance. Initial exercises used body weight or low-resistance elastic bands and focused on functional movements, including:

- Sit-to-stand exercises from a stable chair.
- Supported heel raises.
- Wall push-ups.
- Low step-ups.
- Seated knee extension.
- Standing hip abduction while holding stable support.
- Light elastic-band rowing for older children and adolescents.

Children aged 3–6 years did not perform formal weighted resistance training. Strength-related activity was delivered through play-based movements such as repeated standing, controlled squatting to retrieve toys, reaching and stepping games.

Resistance exercises were performed slowly through a comfortable range of motion. Participants were instructed to breathe continuously and avoid breath-holding. Exercises causing wound pulling, abdominal pressure or graft-site discomfort were discontinued.

## Phase 4: maintenance rehabilitation from 8 weeks to 6 months

From approximately 8 weeks after transplantation, participants with stable clinical status progressed towards regular moderate-intensity aerobic activity. Exercise duration, frequency and complexity were increased gradually according to age and baseline fitness.

Aerobic exercise options included brisk walking, stationary cycling, low-impact aerobic exercise, non-contact ball games, dancing and, after complete wound healing and medical approval, swimming. Jogging, outdoor cycling and more demanding recreational activities were introduced only when balance, strength and clinical status were considered adequate.

Light resistance exercise was performed approximately twice weekly by children aged 7–18 years after medical clearance. Resistance was selected so that all repetitions could be completed with correct technique and without breath-holding, straining or graft-site discomfort. Resistance or exercise complexity was increased only after the participant could complete the prescribed repetitions with good technique during at least two consecutive sessions.

Contact or collision sports and activities associated with a substantial risk of direct abdominal trauma were not included during the six-month study period unless specifically approved by the transplant team.

## Progression algorithm

1. The participant first established regular completion of the prescribed sessions.

2. Session duration was increased before exercise intensity.

3. Only one component—duration, frequency, intensity or exercise complexity—was increased at a time.

4. Duration was generally increased by approximately 5 min per session or by adding one short activity bout.

5. Progression required at least two consecutively tolerated sessions without exercise-related warning symptoms or clinically meaningful next-day fatigue.

6. Moderate-intensity activity was introduced only after adequate tolerance of light-intensity activity.

7. Resistance exercise was introduced only after adequate wound healing and medical clearance.

8. Following an interruption caused by illness or postoperative complications, exercise was restarted at the most recently tolerated level rather than at the planned current target.

9. When excessive fatigue, pain or poor movement quality occurred, the participant returned to the preceding tolerated exercise level.

## Supervision and adherence monitoring

During hospitalization, all rehabilitation sessions were supervised directly by trained nurses or rehabilitation staff. Guardians were encouraged to observe the sessions and practise safe assistance techniques before discharge.

After discharge, guardians supervised younger children, selected enjoyable activities and integrated exercise into daily routines. Children aged 7–12 years participated in selecting activities and completing the exercise record. Adolescents were encouraged to assume increasing responsibility for exercise planning and documentation, with guardian oversight.

Exercise records were reviewed at the 1-, 2-, 4- and 6-month follow-up assessments. Researchers reviewed:

- The number of completed and missed sessions.
- Adherence to prescribed frequency and duration.
- Exercise intensity and tolerance.
- Self-monitoring and record completion.
- Reasons for missed or shortened sessions.
- Exercise-related symptoms.
- Environmental, family or psychological barriers.
- Whether participants or guardians sought professional advice when problems occurred.

The exercise prescription was modified when participants reported poor tolerance, difficulties completing the programme, postoperative complications or new activity restrictions.

# Supplementary Table 1. Rehabilitation exercise prescription according to age and postoperative stage

| **Postoperative stage** | **Age group** | **Main rehabilitation content** | **Prescribed frequency and duration** | **Intensity** | **Criteria for progression** | **Supervision and recording** |
| --- | --- | --- | --- | --- | --- | --- |
| Inpatient phase, generally beginning 24–48 h after transplantation | 3–6 years | Diaphragmatic breathing; ankle pumps; active limb movements; rolling and sitting; standing with assistance; play-based reaching, stepping and short-distance walking | Two to four short sessions daily; initially approximately 5 min per session, progressing to 10–15 min according to tolerance | Very light to light; normal conversation maintained; no visible distress | Able to sit, stand and walk without dizziness, marked fatigue, wound pulling or graft-site pain; stable clinical status; no worsening after activity | Direct supervision by nurses or rehabilitation staff; guardian present when possible; staff documented completion and symptoms |
| Inpatient phase, generally beginning 24–48 h after transplantation | 7–12 years | Breathing and circulation exercises; active range of motion; bed mobility; chair transfer; sit-to-stand; room and corridor walking | Two to four sessions daily; approximately 5–15 min per session | Very light to light; RPE approximately 1–3/10 | Independent transfer or reduced assistance; symptom-free corridor walking; stable clinical observations and movement quality | Direct professional supervision; guardian received instruction; activity recorded by staff |
| Inpatient phase, generally beginning 24–48 h after transplantation | 13–18 years | Breathing exercises; lower-limb circulation exercises; independent bed mobility; standing; repeated sit-to-stand; progressive corridor walking | Two to four sessions daily; approximately 5–15 min per session, progressing according to tolerance | Very light to light; RPE approximately 1–3/10 | Independent mobility; ability to complete walking without excessive fatigue, dyspnoea or wound symptoms; stable postoperative condition | Direct supervision initially, progressing to supervised independent walking; participant encouraged to record symptoms |
| After discharge, postoperative weeks 0–6 | 3–6 years | Comfortable walking with guardian; gentle stretching; movement games; light ball play without running or collision; balance activities near support; routine active play | Two or three 10–15-min activity bouts per day on at least 5 days per week; duration adapted to tolerance | Light; child able to talk and play without visible respiratory distress | Completion without wound discomfort, graft-site pain or prolonged fatigue; gradual improvement in walking and play tolerance | Direct guardian supervision; guardian completed illustrated or written activity record |
| After discharge, postoperative weeks 0–6 | 7–12 years | Walking; gentle flexibility exercises; simple balance exercises; non-contact low-intensity games; light daily activities | Approximately 15–20 min per day initially, progressing to 20–30 min per day on at least 5 days per week; activity could be divided into shorter bouts | Light; RPE approximately 2–3/10; full sentences maintained | Tolerates target duration without warning symptoms; no next-day excessive fatigue; wound healing and graft function remain stable | Guardian supervision, particularly outdoors; child and guardian jointly completed exercise record |
| After discharge, postoperative weeks 0–6 | 13–18 years | Walking; flexibility exercises; low-impact mobility exercises; light stationary cycling only if specifically permitted and safe | Approximately 20–30 min per day on at least 5 days per week; shorter bouts permitted initially | Light; RPE approximately 2–3/10 | Regular completion of prescribed walking; no wound, graft-site or cardiovascular symptoms; clinical clearance for progression | Guardian oversight with increasing self-management; adolescent documented activity and symptoms |
| Postoperative weeks 6–8 | 3–6 years | Faster walking; active play; controlled stepping, reaching, rising from the floor and sit-to-stand games; no formal weighted exercise | Two or three 10–15-min bouts per day on at least 5 days per week | Light, with brief moderate periods during play if well tolerated | Adequate wound healing; stable graft function; no infection, suspected rejection or exercise-related symptoms | Guardian supervision; progression confirmed at clinical review |
| Postoperative weeks 6–8 | 7–12 years | Brisk walking; stationary or safe recreational cycling; non-contact games; flexibility; one set of 8–12 repetitions of sit-to-stand, heel raises, wall push-ups or low step-ups | Aerobic activity approximately 20–30 min per day on at least 5 days per week; strengthening 1–2 days per week | Light to moderate aerobic intensity, RPE approximately 3–4/10; resistance exercises remained light | Can complete current duration while speaking in complete sentences; correct exercise technique; no breath-holding, wound discomfort or prolonged fatigue | Guardian present for strengthening and outdoor activity; exercise log reviewed by research staff |
| Postoperative weeks 6–8 | 13–18 years | Brisk walking; stationary cycling; low-impact aerobic activity; flexibility; 1 set of 8–12 repetitions of body-weight or light elastic-band exercises | Aerobic activity approximately 25–35 min per day on at least 5 days per week; strengthening 1–2 days per week | Light to moderate, RPE approximately 3–4/10 | Completion of two consecutive sessions with correct technique and no warning symptoms; adequate wound healing and medical clearance | Initial guardian or professional instruction; subsequent supervised self-management; participant completed exercise record |
| Postoperative week 8 to month 6 | 3–6 years | Play-based walking, dancing, non-contact ball games, balance and coordination activities; swimming only after wound healing and approval | Two or three 10–15-min structured activity bouts per day on at least 5 days per week, integrated with normal play | Predominantly light to moderate; intensity reduced when the child could not speak normally or showed distress | Increasing activity duration and movement variety without symptoms; stable graft function and clinical status | Guardian supervision and activity selection; guardian recorded adherence and symptoms |
| Postoperative week 8 to month 6 | 7–12 years | Brisk walking; recreational or stationary cycling; swimming after clearance; non-contact games; flexibility; body-weight or elastic-band strengthening | Aerobic activity approximately 20–30 min per day on at least 5 days per week; strengthening approximately twice weekly | Moderate aerobic intensity, RPE approximately 3–5/10; light resistance | Able to complete the prescribed duration and repetitions with correct technique; no exercise-related warning symptoms or next-day functional limitation | Guardian oversight; increasing child participation in planning and recording; review at follow-up |
| Postoperative week 8 to month 6 | 13–18 years | Brisk walking; stationary cycling; low-impact aerobic exercise; swimming after clearance; non-contact recreational sport; light resistance training | Aerobic activity approximately 30–45 min per day on at least 5 days per week; resistance exercise approximately twice weekly | Moderate aerobic intensity, RPE approximately 3–5/10; light resistance allowing controlled repetitions | Regular completion without symptoms; correct technique; stable graft function, wound status and blood pressure; resistance increased only after two well-tolerated sessions | Primarily self-managed with guardian oversight; adolescent maintained exercise log and reported barriers or symptoms |

*Abbreviations: RPE, rating of perceived exertion. Exercise durations represented rehabilitation targets rather than mandatory immediate goals and were reduced or divided into shorter bouts when required.*

# Supplementary Table 2. Exercise-related warning symptoms and protocol modifications

| **Warning symptom or clinical condition** | **Required immediate action** | **Subsequent protocol modification** | **Criteria for restarting or progressing exercise** |
| --- | --- | --- | --- |
| Chest pain, syncope, near-syncope, severe shortness of breath, cyanosis, acute neurological symptoms or severe persistent palpitations | Stop exercise immediately; place the participant in a safe resting position; seek urgent medical assessment | Suspend the rehabilitation programme until the cause has been evaluated | Exercise restarted only after explicit medical clearance, beginning below the previously tolerated level |
| Gross haematuria, new or increasing graft-site pain, graft-area swelling or marked abdominal pain | Stop exercise immediately and contact the transplant team promptly | Suspend all exercise other than essential daily mobility until reviewed | Symptom resolution, clinical assessment and approval from the transplant team |
| Wound bleeding, wound separation, discharge, increasing redness or pain related to the surgical wound | Stop the session and contact the clinical team | Avoid stretching, resistance exercise and movements that tension the wound; suspend structured exercise if advised | Adequate wound healing and clinical clearance |
| Fever, chills or symptoms suggestive of acute infection | Do not initiate exercise or stop the current session; contact the transplant team according to postoperative instructions | Suspend structured exercise during the acute illness; maintain only physician-approved essential mobility | Afebrile status, improvement of systemic symptoms and approval to resume activity |
| Suspected acute rejection, unstable graft function, clinically important reduction in urine output or new oedema | Suspend exercise and inform the transplant team | Programme placed on medical hold; no progression permitted | Stable graft function and explicit clearance from the treating team |
| Uncontrolled hypertension or blood pressure outside the participant’s individualized clinical target | Do not initiate moderate-intensity or resistance exercise; inform the clinical team | Restrict activity to the level specifically approved by the treating physician | Blood pressure controlled and medical clearance obtained |
| Persistent vomiting, diarrhoea, poor oral intake or suspected dehydration | Stop or postpone exercise; restore hydration only according to clinical advice; contact the clinical team when symptoms are clinically significant | Suspend moderate-intensity and outdoor exercise; avoid activity in hot conditions | Adequate hydration, return of oral intake and resolution of systemic symptoms |
| Dizziness, unusual weakness, marked fatigue, nausea or headache during exercise | Stop the session and rest; reassess symptoms | Shorten the next session or return to the previous tolerated duration and intensity | Symptoms resolve promptly and do not recur during routine activity; persistent or recurrent symptoms require clinical review |
| Inability to speak in complete sentences, visibly laboured breathing or RPE above the prescribed range | Reduce speed or resistance; stop the session if symptoms do not improve promptly | Decrease duration or intensity; divide activity into shorter bouts | Able to complete light activity with normal speech and without respiratory distress |
| New musculoskeletal or joint pain, limping, impaired balance or deterioration in exercise technique | Stop the provoking activity | Replace the exercise with a pain-free alternative; reduce resistance or range of motion | Pain-free daily movement and correct technique; persistent pain requires clinical assessment |
| Muscle soreness or fatigue that interferes with normal daily activity on the following day | Do not progress the programme | Repeat or reduce the previous exercise level; increase recovery time between resistance sessions | Normal daily function restored and the previous level is tolerated without recurrent prolonged symptoms |
| Mild transient fatigue that resolves shortly after exercise and does not interfere with daily activity | Rest and document the symptom | Maintain the current level rather than progressing at the next session | Current duration tolerated during at least two consecutive sessions |
| Acute hospitalization, new postoperative complication or physician-directed activity restriction | Suspend the home programme | Follow the activity level prescribed by the treating team | New rehabilitation prescription or explicit approval from the transplant team |
| Interruption of exercise for several days because of illness or complications | Do not resume at the planned higher level | Restart at the most recently tolerated level or lower, with shorter sessions | Gradual progression after at least two symptom-free sessions |
| Contact, collision or combat sports; activities with a substantial risk of direct abdominal impact | Activity not performed as part of the study programme | Substitute non-contact aerobic, coordination or recreational activities | Participation considered only after individualized assessment and specific approval by the transplant team |
| Swimming before complete wound healing or during wound-related or infectious complications | Do not swim | Use land-based walking or mobility exercises as alternatives | Complete wound healing and approval from the transplant team |

# Protocol fidelity

To maintain consistency, the same illustrated exercise guide and standardized verbal instructions were provided to participants and guardians before discharge. The guide described the prescribed activities, age-specific targets, warning symptoms and contact procedures.

At each follow-up, researchers compared the prescribed programme with the exercise record and documented whether the participant had:

1. Completed the prescribed activities.

2. Achieved the recommended frequency and approximate duration.

3. Recorded exercise and symptoms.

4. Modified or stopped exercise appropriately when symptoms occurred.

5. Reported barriers to exercise.

6. Sought professional advice when required.

Any deviations from the standard protocol resulting from postoperative complications, unstable graft function or physician-directed restrictions were recorded as individualized clinical modifications rather than non-adherence.
